# MS2 virus-like particles as a versatile platform for multi-disease vaccines: a review

**DOI:** 10.3389/fimmu.2025.1651594

**Published:** 2025-09-03

**Authors:** Guodong Zhou, Zhili Luo, Zhihui Zhang, Shengliang Cao, Yubao Li

**Affiliations:** ^1^ College of Agriculture and Biology, Liaocheng University, Liaocheng, Shandong, China; ^2^ Shandong Key Laboratory of Applied Technology for Protein and Peptide Drugs, Liaocheng University, Liaocheng, Shandong, China; ^3^ College of Pharmaceutical Sciences and Food Engineering, Liaocheng University, Liaocheng, Shandong, China

**Keywords:** bacteriophage, MS2, coat protein (CP), VLPs, vaccine

## Abstract

The coat protein of the MS2 self-assembles into virus-like particles (VLPs) with a diameter of 26 nm. These VLPs are devoid of the phage genome yet are efficiently recognized by the immune system, eliciting robust humoral and cellular immune responses. The structural characteristics of VLPs position them as a promising platform for the development of vaccines and diagnostic tools. Through genetic engineering, antigenic peptides up to 91 amino acids in length can be densely displayed at the N-terminal β-hairpin (AB loop) of the coat protein. Moreover, the fusion of an exogenous sequence with the 19-nucleotide pac site enables the selective incorporation of heterologous RNA into the VLPs. This feature has facilitated the broad application of VLPs in mRNA vaccine development. In this review, we provide a comprehensive overview of the advancements in MS2 phage coat protein VLP-based vaccine research, with a particular focus on their versatile applications in viral, parasite, chlamydial, and cancer immunotherapy. This work aims to serve as a valuable reference for the continued development of vaccines utilizing MS2 phage coat protein VLPs.

## Highlights

- MS2 VLPs self-assemble into stable icosahedral nanostructures.
- Antigens can be displayed at the AB loop without disrupting assembly.
- RNA cargo can be selectively encapsulated for mRNA vaccine use.
- MS2 VLPs induce strong humoral and cellular immune responses.
- Applicable to viral, bacterial, and cancer vaccine development.

## 1 Introduction

Infectious diseases remain a persistent and evolving threat to global health, with profound implications for both human populations and livestock systems (1, 2). The recent emergence and re-emergence of viral pathogens, including Severe Acute Respiratory Syndrome Coronavirus 2 (SARS-CoV-2), Dengue virus, and Ebola virus, have placed extraordinary pressure on healthcare infrastructures worldwide and triggered widespread socioeconomic disruption (3–5). Alongside these challenges, the continued spread of animal infectious diseases, including foot-and-mouth disease and avian influenza, continues to compromise livestock production and food security worldwide (6, 7). Compounding these issues, the rise of malignant tumors and antimicrobial-resistant bacteria has further increased the complexity of disease prevention and control (8–10).

Inactivated and live attenuated vaccines have been widely deployed to control infectious diseases (6, 11, 12). However, their limited responsiveness to antigenic variability and immune evasion mechanisms poses a major barrier to the development of vaccines against fast-evolving viruses (13, 14). These challenges underscore the urgent need for next-generation vaccines that are not only highly efficacious but also amenable to rapid deployment and broad accessibility during infectious disease outbreaks (15–17). The design of effective vaccines is deeply informed by the structural and immunological properties of viruses. Structural characteristics such as nanoscale dimensions, ordered symmetry, and repetitive antigenic arrays are key determinants in how the immune system recognizes and responds to viral pathogens (18). Mimicking these characteristics, VLPs, self-assembled from viral structural proteins, have emerged as a robust platform for antigen presentation (18, 19).

VLPs recapitulate the external architecture of native viruses but lack genetic material, thereby ensuring safety while inducing strong humoral and cellular immune responses (20, 21). Bacteriophage-based VLP systems provide several distinct advantages over other platforms, including robust self-assembly, genetic tractability, cost-efficiency, and suitability for high-yield, large-scale production (22). Notably, the MS2, an icosahedral single-stranded RNA (ssRNA) virus of the *Leviviridae* family, has been widely explored as a modular scaffold for vaccine design (22–24). The CP of MS2 spontaneously forms icosahedral nanoparticles consisting of 180 monomers, preserving the native capsid architecture with high fidelity (25). Structural studies have demonstrated that its N-terminal β-hairpin, known as the AB loop, can tolerate the insertion of heterologous peptides up to 91 amino acids without disrupting particle assembly (26). The targeted genetic modification of the AB loop has facilitated the presentation of heterologous epitopes, thereby advancing the development of vaccine candidates targeting a wide array of pathogens, including viruses, bacteria, parasites, and malignant cells (22–24). Furthermore, the dimeric form of the MS2 CP exhibits high-affinity binding to stem-loop RNA structures called pac sites, which directs the encapsidation of RNA cargo with sequence specificity (27). This property offers a unique opportunity to leverage the MS2 platform for mRNA vaccine delivery, expanding its utility beyond peptide-based immunization (28).

MS2 VLPs have gained increasing attention as a versatile and promising platform for vaccine development. This review highlights recent progress in the design and biomedical application of MS2 VLP-based vaccines, with an emphasis on their roles in infectious disease prevention and cancer immunotherapy across human and veterinary medicine. These insights may provide a conceptual and technological basis for the rational design of next-generation vaccine platforms.

## 2 Advantages of bacteriophage VLPs

VLPs are nanoscale protein assemblies that structurally resemble native viruses. They are typically formed through the self-assembly of viral capsid proteins, although non-viral proteins with similar architectural properties can also serve as building blocks (20, 21, 29). This assembly process is governed by non-covalent interactions—such as hydrophobic forces, hydrogen bonding, and electrostatic attraction—which enable the spontaneous formation of stable, highly ordered particles (29). VLPs can be engineered to present antigenic proteins on their surface with defined spatial orientation and stoichiometry, thereby promoting efficient and targeted immune activation (19, 29). Diverse VLP platforms have been developed using self-assembling proteins derived from bacteriophages, plant and animal viruses, as well as synthetic scaffolds such as ferritin, small heat shock proteins, and E2 acyltransferase (29). These systems have demonstrated considerable potential for enhancing immune responses and for the prophylactic or therapeutic targeting of a broad range of infectious diseases (30).

Among these VLP platforms, bacteriophage-derived proteins are particularly promising due to their natural self-assembly properties, structural controllability, rapid production timelines, and cost-effectiveness (22). Phage VLPs exhibit excellent biocompatibility and stability, efficiently presenting antigens and stimulating immune responses without the need for additional adjuvants (22, 30). For instance, the T4 phage capsid, composed of 870 copies of small outer capsid proteins (Soc) and 155 copies of highly immunogenic outer capsid proteins (Hoc), can display up to 860 copies of the HIV - 1 gp41 envelope protein (30, 31). This density is 60 – 120 times greater than that observed on the surface of natural HIV virions. Similarly, capsid proteins from Qβ, MS2, and λ phages have been shown to self-assemble into nanoparticles with the theoretical capacity to present over 180 antigenic epitopes (30), though this capacity is often influenced by the physical limits of insertion tolerance and particle stability, which remain areas of active investigation.

**Figure 1:**
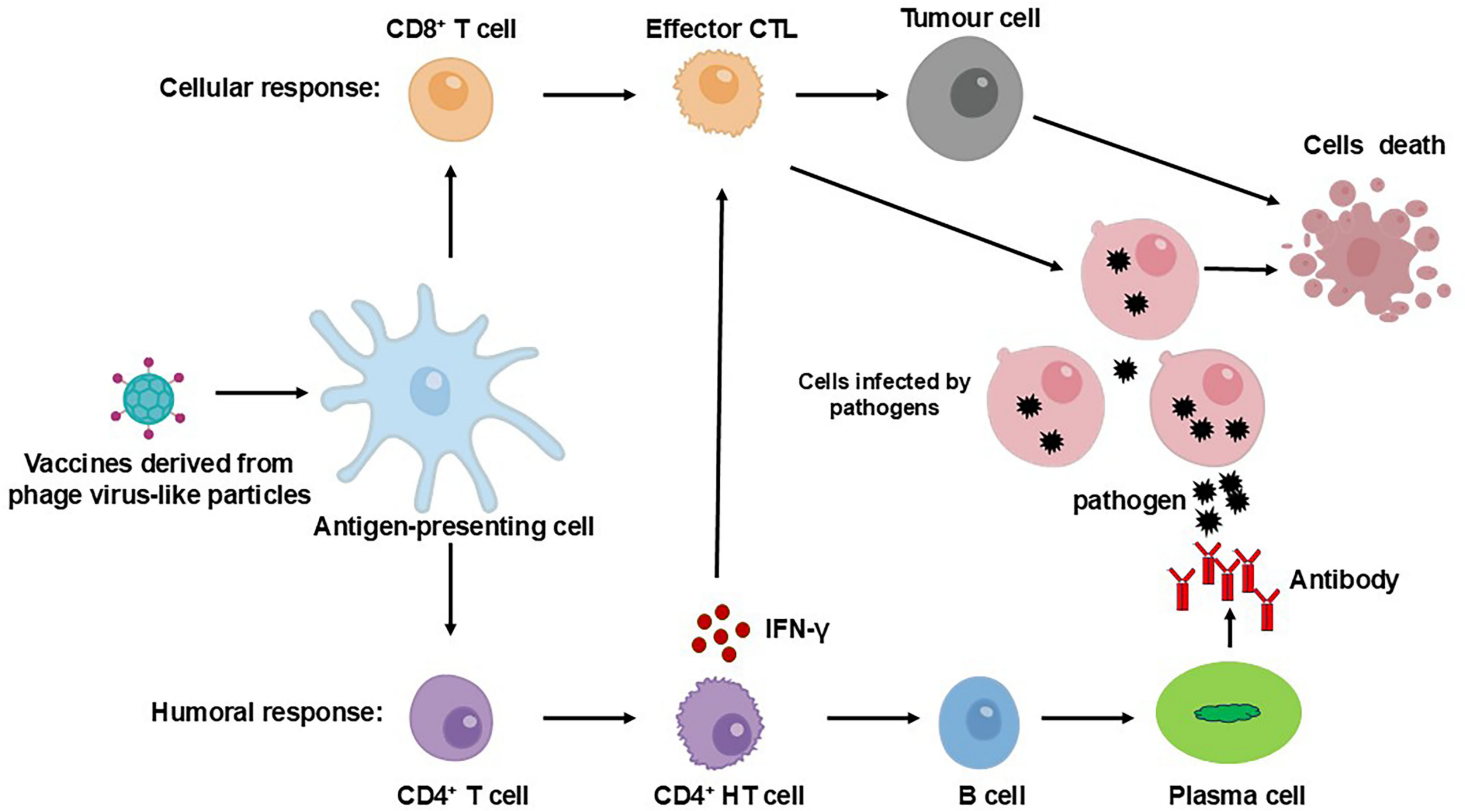
Adaptive immune activation induced by phage VLP-based vaccines. Following vaccination, phage VLP-based vaccines are captured by APCs, such as dendritic cells. These vaccines are processed and presented via MHC-I and MHC-II pathways, which are critical for the activation of CD8+ and CD4+ T cells, respectively. To induce a humoral immune response, B cells interact with CD4+ T helper (TH) cells. Through B cell receptors, B cells internalize the VLP-based vaccine, and subsequent interaction with CD4+ TH cells stimulates plasma cells to secrete a sufficient quantity of IgG antibodies, while also generating B memory cells. For cellular immunity, immature CD8+ cytotoxic T lymphocytes (CTLs) proliferate and differentiate into effector CTLs and specific memory CTLs. Effector CD4+ TH cells aid in CTL activation through the secretion of cytokines.

The immunological benefits of phage-derived VLPs arise not only from their high antigen density but also from their favorable biophysical properties. Nanoparticles in the range of 20 to 200nm fall within the optimal size window for efficient uptake by antigen-presenting cells (APCs), thereby enhancing antigen internalization, processing, and presentation to T cells (29, 30, 32). The virus-like morphology of phage VLPs, including features such as shape, surface charge and hydrophobicity, further facilitates recognition by the innate immune system and promotes downstream adaptive responses (**Figure 1**) (30). Critically, the repetitive and high-density display of antigens on VLP surfaces promotes efficient cross-linking of B cell receptors, leading to robust B cell activation and strong humoral immune responses (33). Moreover, during the phage lysis of its host bacteria, lipopolysaccharides may be carried on the surface, which strongly activates TLR4 and bolsters adaptive immune responses (34). While these properties are frequently cited as key advantages, the relative contribution of each to overall immunogenicity remains to be quantitatively defined, and systematic comparisons across different VLP platforms are still lacking.

While bacteriophage VLPs often generate robust immune responses even in the absence of added adjuvants, co-administration with adjuvants has been shown to significantly amplify their immunogenicity (35). Nonetheless, despite these advantages, challenges remain. These include structural constraints on antigen insertion sites, batch-to-batch reproducibility in large-scale production, and control over unintended immune activation, which warrant continued refinement of VLP design strategies. Overall, both viral and non-viral VLPs offer versatile platforms for vaccine development, particularly in the context of infectious disease prevention, cancer immunotherapy, and the exploration of novel immunostimulatory designs (**Figure 1**). The MS2, a classic example of VLPs platform, has gained increasing attention in recent years for its potential in innovative vaccine development (23, 26, 28).

## 3 MS2 bacteriophage: evolving from bacterial therapy to a versatile vaccine platform

**Figure 2:**
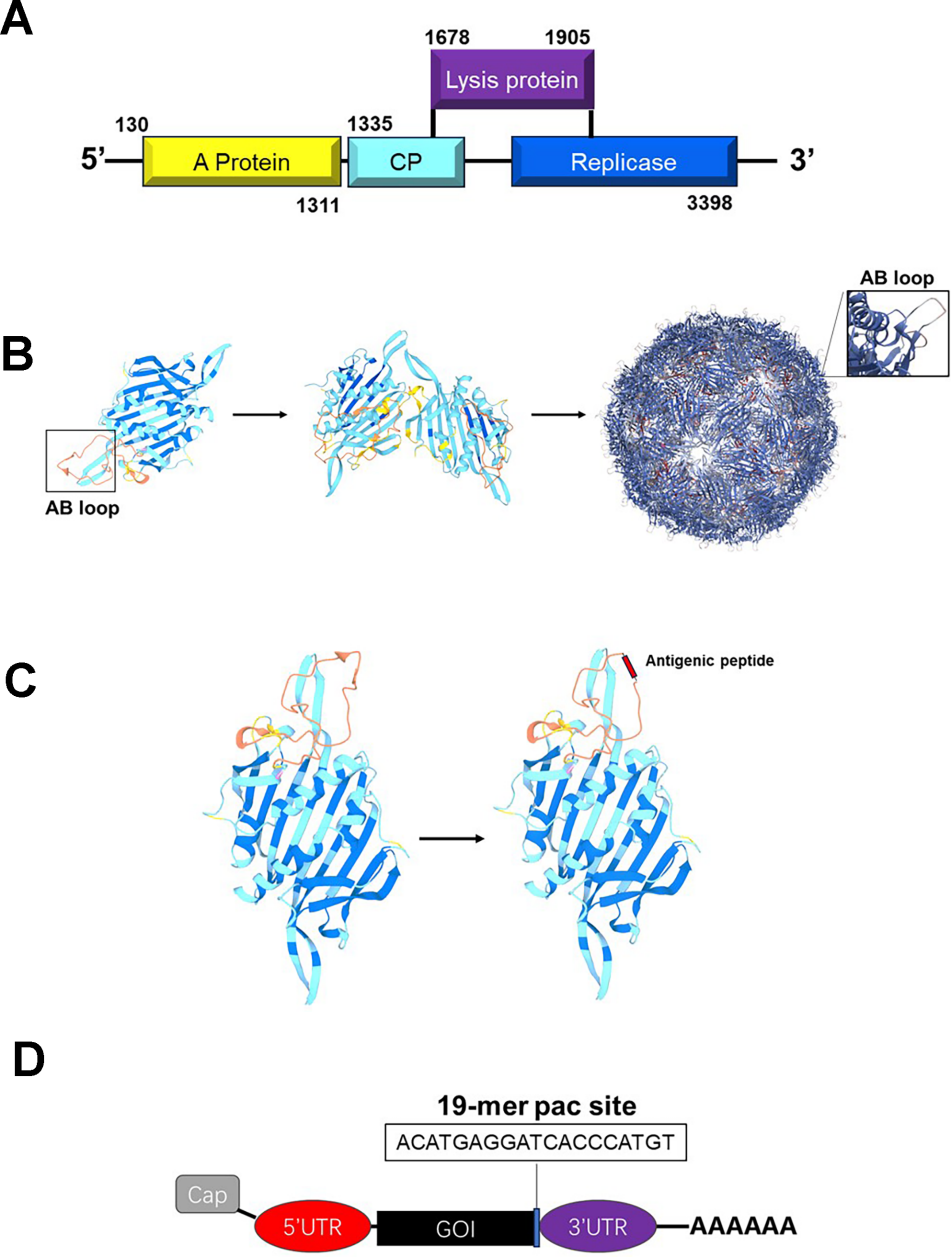
Structure of phage MS2 and its basis for vaccine development. **(A)** The genome of phage MS2 encodes four essential proteins: the capsid protein (CP, the major protein), the maturation protein (protein A), the replicase (RNA polymerase, necessary for genome replication), and the lysis protein. **(B)** The capsid protein of phage MS2 can self-assemble into VLPs, providing a foundation for vaccine development. **(C)** The AB loop region of the MS2 capsid protein can be substituted with antigenic peptide sequences, enabling the display of specific antigens. **(D)** Integrating the pac site into the target mRNA allows for efficient encapsulation of the mRNA.

MS2 is an icosahedral RNA bacteriophage with a triangulation number of T = 3, and its crystallographic structure has been resolved at a 2.8 Å resolution (22–24). The genome of bacteriophage MS2 consists of a positive-sense single-stranded RNA molecule of 3569 nucleotides, encoding four proteins: the major coat protein, the maturation protein (A-protein), the replicase (an RNA polymerase essential for genome replication), and the lysis protein (**Figure 2A**) (36). It primarily infects *Escherichia coli* and other members of the Enterobacteriaceae family (37). During natural infection, capsid assembly is initiated through CP dimerization and specific binding to a characteristic RNA hairpin structure, termed the pac site, within the viral genome (27). Remarkably, when the *cp* gene is expressed independently in bacterial or yeast systems, CP monomers can spontaneously self-assemble into VLPs that are morphologically similar to native virions but lack genomic RNA and other structural components (**Figure 2B**) (23, 26, 28).

Structural studies have revealed that the assembly mechanism of MS2 VLPs is dependent on conformational dynamics of the unstructured FG loop, which connects the F and G β-strands within each CP monomer (24, 36, 38). This dynamic region enables CP to adopt three distinct conformations, designated A, B, and C (24, 38). These conformations subsequently form asymmetric A/B dimers or symmetric C/C dimers (24, 38). Ultimately, 90 CP dimers (comprising 60 A/B-type and 30 C/C-type dimers) organize into a complete icosahedral VLP (24, 27, 36). This unique dimer distribution pattern effectively compensates for the absence of the maturation protein by enhancing capsid stability through structural complementarity. Building upon these findings, the CP of MS2 serves as an effective nanoscale scaffold for the high-density display of antigenic peptides (**Figure 2C**).

Among its most notable features, the N-terminal β-hairpin structure (the AB loop) demonstrates considerable insertional flexibility, tolerating foreign peptide sequences—including viral and non-viral antigens—up to 91 amino acids in length (26). Although high insertion tolerance facilitates the development of immunogen-rich VLPs, structural perturbations induced by large inserts may compromise native symmetry and particle integrity, a challenge that has been addressed through rational engineering approaches. Thus, while the AB loop presents a valuable insertion site, its use requires careful consideration of structural and functional trade-offs.

In addition to surface presentation, MS2 VLPs can encapsidate exogenous RNA through engineered pac site interactions. The 19-nucleotide pac motif retains its CP-binding capability when fused to heterologous transcripts, enabling selective packaging into MS2-derived VLPs (39). This discovery led to the development of pac site-based RNA protection strategies against RNase degradation (**Figure 2D**). However, nonspecific encapsidation of host RNA remains a critical limitation, particularly for therapeutic applications. To address this, co-expression strategies have been explored. For example, a single-plasmid system encoding CP, maturation protein, and pac-tagged target RNA within a single transcriptional unit was shown to improve packaging specificity by aligning RNA: CP stoichiometry (40). A dual-plasmid strategy further enhanced RNA loading by separately expressing pac-tagged RNA and structural proteins, allowing independent modulation of plasmid copy numbers and antibiotic selection (41).

Importantly, sequence engineering of the pac motif, particularly the C5 variant in which the fifth uridine is substituted with cytidine, has been shown to increase the binding affinity between CP and target RNA by approximately 6- to 50-fold, thereby enabling the encapsidation of transcripts up to 2248 nucleotides in length (42). In response to the metabolic burden associated with the dual-plasmid system, a single-plasmid dual-expression system was engineered (43). This system employed a modular cloning approach, where the CP/maturation protein and the target RNA, containing triple pac sites, were strategically positioned at separate loci. As a result, efficient packaging of RNA cargos up to 3 kb was achieved, thereby significantly increasing the payload capacity of the system. These optimizations substantially expanded the payload potential of the MS2 system, although the impact of pac site multiplicity and sequence context on packaging fidelity remains an area for further study.

Together, these structural and functional attributes establish MS2 as a modular VLP platform, capable of both high-density antigen display and programmable RNA encapsidation. While the system has proven versatile in preclinical applications, continued refinement will be essential to overcome structural constraints and improve precision in molecular cargo selection. In the following sections, we systematically review the application strategies and recent advances in the use of MS2 coat protein nanoparticles for vaccine development.

## 4 Applications of MS2 virus-like particles in vaccines against viral infections

### 4.1 Human immunodeficiency virus

The self-assembled VLPs formed by the MS2 CP offer several advantages for peptide display. However, studies have shown that peptide insertion, even within surface-exposed loops, can interfere with proper protein folding and VLP assembly. For example, insertion of peptides derived from the V3 loop of HIV gp120 and the ECL2 loop of the HIV co-receptor CCR5 into MS2 CP disrupted capsid formation (44). Notably, engineering the CP as a single-chain dimer, by genetically linking two CP monomers, mitigated these folding defects and enabled the successful display of V3 and ECL2 peptides on the surface of MS2 VLPs. Immunization of mice with these constructs induced specific IgG antibodies against the displayed peptides, which were also capable of recognizing the full-length VP3 protein.

More recently, the HIV - 1 FP8 epitope, which is critical for VRC34.01 neutralization, was targeted by inserting the FP8 sequence into MS2 CP dimers, generating MS2-FP8 VLPs (45). As a comparator, synthetic FP8 peptides bearing a free cysteine were chemically conjugated to Qβ VLPs using the crosslinker SMPH, generating Qβ-FP8 conjugate particles. Both MS2-FP8 and Qβ-FP8 VLPs bound VRC34.01 with high affinity, confirming the preservation of critical FP8 conformational epitopes. Although MS2-FP8 induced lower antibody titers than Qβ-FP8, antibodies from both platforms recognized full-length BG505 DS-SOSIP trimers. The observed differences are likely due to variations in antigen density, with MS2 displaying approximately 90 copies of FP8 per particle, compared to over 200 copies on Qβ. Importantly, a heterologous prime-boost regimen (MS2-FP8 prime, Qβ-FP8 boost, BG505 DS-SOSIP boost) elicited superior HIV - 1 neutralizing antibody responses compared to homologous immunization strategies. Alternating VLP platforms likely reduces anti-carrier interference, providing a valuable strategy for optimizing vaccine efficacy.

In addition to enhanced peptide tolerance, single-chain dimer MS2 VLPs retain the capacity to encapsidate their own mRNA, supporting applications in RNA vaccine development. Since prokaryotic mRNA lacks the 5′ cap and 3′ polyA tail essential for eukaryotic translation, adapting MS2 VLPs for functional mRNA delivery remains challenging. A yeast-based system was established to produce MS2 VLPs encapsidating human growth hormone mRNA, achieving efficient translation both *in vitro* and in mammalian cells (46). Building on this strategy, MS2 VLPs encapsidating gag mRNA (1544 nt) were developed as RNA vaccine candidates against HIV - 1, successfully inducing antigen-specific humoral responses in mice (46). The conserved characteristics of Gag proteins suggest that these vaccines could be promising tools for viral control.

### 4.2 Human papillomavirus

HPV is a double-stranded DNA virus that primarily infects the skin and mucosal epithelia, with sexual contact being the primary mode of transmission (47). The virus is classified into high-risk and low-risk categories, with high-risk genotypes being significantly correlated with the onset and progression of cervical cancer (48). Its capsid is composed of two structural proteins, L1 and L2, with L1 serving as the primary scaffold of the capsid architecture (49, 50). When expressed exogenously, L1 self-assembles into VLPs that closely mimic the native virions (51). These L1-based VLPs form the basis of current HPV vaccines, such as Cervarix, Gardasil-4, and Gardasil-9, which have proven effective in inducing high-titer neutralizing antibodies (52). However, the protective efficacy of these vaccines is largely confined to the HPV types included in the vaccine, with limited cross-protection, especially in immunocompromised individuals. As a result, there has been growing interest in targeting the minor capsid protein L2, which contains highly conserved epitopes that could provide broader protection across diverse HPV types (50).

It has been demonstrated that the N-terminal 170 amino acids of L2 are particularly potent in inducing neutralizing antibodies that remain conserved across different HPV types (53). These antibodies not only inhibit homologous HPV infections in animal models but also exhibit cross-neutralization against heterologous HPV strains, suggesting that L2 could play a crucial role in mediating broad-spectrum immune protection. The N-terminal region of the MS2 CP was modified by inserting four L2-derived peptides from HPV16 (aa 20 - 29, aa 17 - 31, aa 14 - 40, and aa 14 - 65), resulting in the formation of self-assembling MS2-derived nanoparticles (54). Three of these constructs successfully self-assembled into particles and exhibited strong immunogenicity, inducing high-titer antibodies against L2 in immunized mice. Cross-reactivity assays revealed that the antibodies generated by these MS2-L2 nanoparticles had a broader range of recognition across multiple HPV isolates compared to existing vaccines. Notably, MS2-L2 (17 - 31) construc conferred complete protection against high-dose HPV16 infection in mice and effectively neutralized multiple HPV pseudoviruses. Spray-drying of MS2-L2 (17 – 31) formulation into a dry powder was found to enhance their thermostability, permitting storage at room temperature for up to 34 months (55, 56). Despite extended storage, the particles retained their protective efficacy against HPV16 infection in immunized mice.

In addition to displaying single epitopes, other studies have utilized the MS2 VLPs platform to present multiple L2 epitopes from different HPV types, aiming to expand the immune coverage of these vaccines. Successful insertion of L2 tandem peptides from HPV16 and HPV31, along with consensus L2 sequences from 19 high-risk and 4 low-risk HPV types, into the N-terminal of MS2 CP resulted in the generation of five distinct chimeric nanoparticles (57). Immunization with these multi-epitope constructs induced strong IgG responses in mice, particularly against HPV16, HPV31, and the consensus L2 (69 - 86) peptide, confirming that displaying multiple epitopes does not diminish the immunogenicity of individual peptides. It has been demonstrated that MS2 - 31/16L2 and mixed MS2-L2 nanoparticles provided protection against six HPV types, with efficacy comparable to Gardasil-9. These results suggest that MS2-based multi-epitope nanoparticles represent a promising strategy for developing HPV vaccines with enhanced cross-protection.

Building upon the expanded protection offered by multi-epitope constructs, additional research has explored the potential of MS2-L2 nanoparticles in vaccine formulation (58). In a mouse model, immunization with MS2-L2 nanoparticles, combined with mucosal adjuvants such as cholera toxin and monophosphoryl lipid A, induced high-titer serum IgG responses and provided broad protection against 11 HPV types in cervical and oral pseudovirus challenge experiments. This approach covered approximately 95% of cervical cancer-causing and 99% of head and neck cancer-causing HPV types, with protection against certain strains (HPV35 and HPV 39) even superior to Gardasil-9. Furthermore, mixed MS2-L2 nanoparticles were formulated into a powder vaccine using spray-drying technology, which significantly improved the vaccine’s thermal stability and allowed for storage at room temperature for up to 60 days without adjuvants (59). This development represents an important step toward creating HPV vaccines with enhanced accessibility, particularly for global distribution.

### 4.3 Influenza virus

Vaccination with seasonal influenza vaccines or natural infection predominantly induces immune responses directed against the globular head domain of the viral hemagglutinin (HA) protein (60, 61). However, this immunodominant region is highly susceptible to antigenic drift, enabling viral immune evasion and necessitating frequent reformulation of vaccines (61). To address these limitations, MS2 VLPs have been engineered as modular antigen presentation platforms (62). By incorporating an AviTag sequence into the capsid protein of MS2, site-specific biotinylation was achieved, enabling high-affinity biotin–streptavidin interactions for the directional display of HA antigens. Two MS2-based constructs were developed: one displaying HA in its conventional orientation with the immunodominant head domain exposed, and another in an inverted configuration, where HA was anchored via an anti-head Fab fragment to expose the conserved stem domain. Immunogenicity studies demonstrated that the inverted nanoparticles elicited anti-stem antibody titers that were 5- to 10-fold higher than those induced by conventional constructs. Moreover, antibodies generated by the inverted nanoparticles exhibited broader cross-reactivity and conferred significantly enhanced protection in the context of high-dose viral challenge. This orientation-based antigen display strategy effectively mitigates the structural shielding of conserved epitopes by the HA head and offers a promising avenue for the development of broadly protective influenza vaccines, as well as vaccines targeting other variable pathogens.

To investigate the potential of MS2 VLPs to elicit durable immune responses, a candidate influenza vaccine, PR8HA-MS2, was developed using an MS2-based VLP platform. These particles, approximately 67 nm in diameter, display the HA antigen from the A/Puerto Rico/8/1934 (PR8) strain in a conventional outward-facing orientation at high surface density. Immunogenicity was evaluated in a ferret model, where a single intramuscular dose of PR8HA-MS2 formulated with either AddaVax or Quil-A adjuvant elicited robust neutralizing antibody responses within three weeks, with geometric mean titers ranging from 853 to 942 substantially higher than those observed in the unadjuvanted group and placebo controls. Upon homologous viral challenge, vaccinated animals exhibited significantly reduced viral replication in the nasal mucosa and a shortened viral shedding duration of only three days. Long-term serological analysis showed that neutralizing antibody titers in adjuvanted groups remained stable for over 3.5 years (20 – 183 weeks), and were further boosted following secondary immunization, indicating a strong and sustained memory response.

### 4.4 Severe acute respiratory syndrome coronavirus 2

SARS-CoV-2 is a highly contagious coronavirus that causes severe respiratory illness, multi-organ damage, and even death, particularly threatening immunocompromised individuals (63). The spike (S) protein on the surface of SARS-CoV-2 is responsible for receptor recognition and membrane fusion, with the receptor-binding domain on the S protein being a critical vaccine target that can trigger the production of neutralizing antibodies (64). Given the inherent advantages of VLPs in antigen presentation, a study utilized gene fusion to insert an AviTag site onto the surface of MS2 dimers (65). This allowed high-density streptavidin-mediated conjugation of prefusion-stabilized S proteins, yielding MS2-based constructs termed S2Pro and S6Pro, each approximately 30 – 50 nm in diameter (65). These constructs were shown to have retained the proper conformation of the S protein as well as its binding capacity to the ACE2 receptor, as revealed by further characterization. In animal experiments, hamsters developed high-titer neutralizing antibodies after a single dose of immunization, and no live virus was detected in lung tissues after challenge infection, demonstrating excellent protective efficacy.

Recognizing the limitations of targeting variable epitopes, a parallel approach utilized MS2 virus-like particles to construct a vaccine directed against the conserved S2 subunit of the coronavirus spike protein (66). The S2 subunit is less prone to antigenic drift compared to the highly variable S1 region and is therefore an attractive candidate for universal coronavirus vaccine development. Using a similar AviTag–streptavidin system, biotinylated S2 subunits were conjugated onto MS2 VLPs, yielding particles approximately 43 nm in diameter that displayed ~30 copies of S2 per particle. Dynamic light scattering and cryo-electron microscopy confirmed the structural stability of the particles and preservation of the native antigenic conformation of the S2 protein. In Syrian hamsters and BALB/c mice, both single- and triple-dose immunization regimens with S2-decorated nanoparticles formulated with AS03 and poly(I:C) adjuvants elicited strong, durable, and cross-reactive IgG responses against a broad panel of coronaviruses, including SARS-CoV-2 (ancestral and Beta, Delta, Omicron variants), SARS-CoV-1, pangolin CoV (Pg-CoV), and endemic human coronaviruses such as HKU1 and OC43. Upon challenge, immunized animals exhibited significant reductions in viral loads in the respiratory tract, including a >6,000-fold decrease in Omicron BA.1 lung titers and complete inhibition of Pg-CoV replication. Mechanistically, the multivalent display of the conserved S2 antigen enhanced B cell receptor cross-linking and circumvented the immune evasion challenges associated with mutations in the S1 domain, particularly within the RBD. Further optimization revealed that while a three-dose regimen maximized antibody titers and protective efficacy, even a single dose was sufficient to fully suppress replication of adapted viral strains in murine models. Collectively, these findings underscore the versatility and promise of MS2 VLPs as a modular platform for developing next-generation pan-coronavirus vaccines that target conserved structural epitopes.

### 4.5 Zika virus

ZIKV is primarily transmitted by mosquitoes and poses significant risks to pregnant women, as it can lead to severe birth defects such as microcephaly and neurological complications, including Guillain-Barré syndrome (67). The ZIKV envelope protein has been shown to contain several potential B-cell epitopes, with key regions located at residues 241 – 259, 294 – 315, 317 – 327, 346 – 361, 377 – 388, and 421 – 437 (68–71). These findings provide a promising foundation for the development of peptide vaccines targeting ZIKV infection.

To explore this, MS2 VLPs were used as a carrier for displaying the B-cell epitope (amino acids 377 - 388) from the ZIKV envelope protein at the N-terminus of the MS2 CP through gene insertion (72). This led to the successful construction of MS2-Zika-E377–388 nanoparticles. Transmission electron microscopy and ELISA assays confirmed correct assembly and efficient epitope presentation. In mouse immunization studies, MS2-Zika-E377–388 nanoparticles induced specific IgG antibodies against ZIKV, with a geometric mean titer of approximately 10³. However, their neutralizing activity against ZIKV was relatively weak when administered alone. In contrast, Qβ VLPs, which displayed the epitope via chemical conjugation and had a higher epitope copy number (180 copies), exhibited stronger immunogenicity.

Additionally, combining MS2-Zika-E377–388 nanoparticles with Qβ VLPs displaying different epitopes slightly enhanced the neutralizing response, although overall protection remained limited. These results suggest that MS2 VLPs could serve as a potential platform for ZIKV vaccine development, but further optimization of epitope presentation strategies or combination with other platforms is necessary to improve immunogenic efficacy.

### 4.6 Foot and mouth disease

FMD is a highly contagious viral disease caused by the foot-and-mouth disease virus (FMDV), which poses a significant threat to livestock such as cattle, pigs, and sheep, leading to high mortality in young animals and reduced productivity in adults (73). Traditional inactivated vaccines have played a crucial role in controlling outbreaks, but they have notable limitations, including the risk of virus leakage during production, poor heat stability, and a short duration of protection (74). Therefore, there is a pressing need to develop safer, more stable, and highly effective vaccines.

In response to these challenges, a novel vaccine candidate was developed using the MS2 VLPs platform, which was engineered to display EP141 - 160, a key neutralizing epitope derived from the VP1 capsid protein of FMDV (75). The sequence encoding this epitope was precisely inserted into the AB-loop region of the MS2 CP. After immunization of mice, the vaccine induced high titers of specific IgG antibodies, comparable to those produced by traditional inactivated vaccines. Further challenge experiments in guinea pigs and pigs demonstrated that the CP-EP141–160 nanoparticles effectively induced the production of neutralizing antibodies and provided clinical protection in 65% of guinea pigs and 60% of pigs.

Subsequent research focused on another B cell epitope, amino acids 131 - 160, from the FMDV VP1 protein, which includes the critical G-H loop and its flanking sequences (76). The recombinant nanoparticles vaccine showed significantly higher levels of IgG antibodies and better cellular immune responses than traditional peptides and commercial vaccines (77). Structural analysis indicated that the nanoparticles retained a circular conformation, enhancing both B cell and T cell activation. However, the study did not include challenge protection experiments, and the practical application of this vaccine remains to be further investigated.

## 5 Plasmodium Falciparum

Malaria, an endemic disease in tropical and subtropical regions, is transmitted by vectors and caused by protozoan parasites of the genus *Plasmodium (* 78). Fever, chills, and headache are the most frequently observed symptoms in infected individuals, and the disease is known for its substantial mortality rate. Although antimalarial drugs such as artemisinin derivatives remain the mainstay of treatment, vaccines are also recognized as a critical strategy for malaria control (78, 79). RTS,S/AS01 and R21/Matrix-M, the vaccines currently approved for malaria, have been deployed in several countries and regions worldwide (80). However, their protective efficacy remains suboptimal. Liver stage antigen-1 (LSA - 1), a ~200 kDa polypeptide expressed on the surface of *P. falciparum* schizonts during the liver stage, contains non-repetitive regions at its N- and C-termini encoding 14 – 24 amino acid peptides capable of eliciting IL - 10, TNF-α, IFN-γ, and CD8^+^ T cell responses. Based on this, the Th1 epitope located within the N-terminal non-repetitive region was fused to the MS2 CP to generate a chimeric phage-like particle, and its immunogenicity was evaluated in BALB/c mice (81). Comparative studies showed that native MS2 VLPs predominantly induced humoral and cellular immune responses skewed toward a Th2 cytokine profile, whereas the Th1 peptide-bearing chimeric particles elicited a primarily Th1-type immune response following immunization.

In a separate study targeting the blood-stage invasion process, researchers focused on RH5, a highly conserved adhesin essential for P. falciparum red blood cell invasion. A monoclonal antibody capable of fully inhibiting RH5-mediated entry was employed in an affinity screening of a random peptide library displayed on MS2-based nanoparticles (82). The peptides (SAIKKPVT) identified through this process were then used as immunogens to produce specific antisera, which were subsequently evaluated for their ability to block parasite invasion. Immunization with MS2-derived particles displaying this peptide elicited potent anti-RH5 antibody responses, and the resulting antisera inhibited erythrocyte invasion by over 90% *in vitro*. These findings support the utility of RNA phage capsid display systems for epitope screening and validate MS2 coat protein-based nanoparticles as a versatile platform for peptide-based malaria vaccine development.

## 6 Chlamydia trachomatis

*Chlamydia trachomatis* (Ct) exhibits multiple serotypes primarily determined by variations in the amino acid sequences of the major outer membrane protein (MOMP) on the surface of the elementary body (83). Conserved epitopes within variable domain 4 (VD4) of MOMP are recognized as critical targets for inducing broad-spectrum neutralizing antibodies (84). Efforts to develop vaccines targeting MOMP have been hindered by the limited immunogenicity of recombinant proteins and the challenges in extracting native proteins. While an engineered extVD4 multivalent vaccine, combined with the CAF01 adjuvant, has demonstrated some cross-protection against Ct serotypes D, E, F, and G, the complexity of the antigenic structure highlights the necessity of accurately mimicking the natural epitope conformation (84).

To enhance vaccine efficacy, the MS2 VLPs platform has been employed to develop vaccines targeting the core neutralizing epitope (TTLNPTIAG) in MOMP-VD4 (85). Two strategies were applied to identify potential immunogens: The first involved affinity selection using the broadly neutralizing E4 monoclonal antibody, leading to the isolation of a peptide-presenting construct (HMVGSTKWTN). Although this construct elicited antibodies that weakly recognized the Ct elementary body, competitive binding assays indicated preferential recognition of the native VD4 epitope. The second strategy utilized site-directed insertion, expressing the TTLNPTIAG peptide and its C-terminal mutant TTLNPTIAGA on the exposed loop of the MS2 CP. Both resulting MS2-based constructs elicited VD4-specific IgG responses in mice, with the native sequence construct (MS2-VD4.A) providing superior protection in a vaginal challenge model.

Together, these findings support the potential of MS2-based nanoparticles as a modular platform for presenting conformational Ct epitopes and advancing peptide-based vaccine strategies with both broad-spectrum coverage and structural fidelity.

## 7 Applications of MS2 virus-like particles in cancer immunotherapy

### 7.1 Prostate cancer

Prostate acid phosphatase (PAP) is a widely expressed antigen in prostate tissue (86). Studies have demonstrated that PAP, whether in the form of a protein, peptide, or DNA, can elicit a specific cellular immune response against prostate cancer and even promote tumor regression (86–88). Moreover, prior research has highlighted the potential of granulocyte-macrophage colony-stimulating factor (GM-CSF) as an effective adjuvant, with proven applications in the development of prostate cancer vaccines (89). Based on these findings, an expression vector was designed to incorporate human or mouse-derived PAP alongside mouse GM-CSF mRNA (90). The target mRNA is specifically packaged into MS2 CP via the pac site, yielding MS2 VLP-mRNA vaccines that are resistant to nuclease degradation. Functional validation confirmed that this packaging approach protected the mRNA from degradation for at least 18 hours, facilitated macrophage uptake, and supported efficient expression of heterologous proteins such as GFP. In animal models, the vaccine elicited robust humoral and cellular immune responses, including activation of CD4^+^ T cells, cytotoxic T lymphocytes (CTLs), and a mixed Th1/Th2 response, along with enhanced maturation of dendritic cells. Notably, the proportion of regulatory T cells remained stable, indicating that antigen-specific immunity was achieved without perturbing immune homeostasis. In prophylactic models, the vaccine provided complete protection against tumor formation in mice, and in therapeutic models, it significantly delayed prostate tumor growth, outperforming traditional PAP DNA vaccines and single-antigen strategies. Additionally, human PAP vaccines exhibited stronger immunogenicity than mouse PAP vaccines, suggesting that heterologous mRNA more effectively breaks immune tolerance.

### 7.2 Breast cancer

Breast Cancer (BC) is one of the leading causes of cancer-related mortality in women (91). Given the pivotal role of breast cancer stem cells (BCSCs) in BC recurrence and metastasis, a novel vaccine, AX09, has been developed based on the MS2 VLPs platform (92). This vaccine targets the third extracellular domain (ECD3) of the glutamate/cysteine transporter subunit (xCT), which is highly expressed on BCSCs (93). The resulting nanoparticles demonstrated excellent thermal stability and long-term storage properties. Immunization studies in mice showed that AX09 induced high titers of functional anti-xCT antibodies across various genetic backgrounds (BALB/c, C57BL/6, CD - 1). These antibodies effectively bound both linear and native conformations of the xCT-ECD3 epitope, and specifically recognized xCT expressed on tumor cell surfaces, without causing toxicity in normal brain tissue. Functional studies revealed that AX09-induced antibodies inhibited tumor spheroid formation, self-renewal, cysteine uptake, and cell migration, while significantly increasing intracellular reactive oxygen species levels, indicating effective blockade of xCT function. Additionally, AX09 significantly reduced lung metastasis and delayed primary tumor growth across various BC models, including TUBO cell intravenous injection, 4T1 spontaneous metastasis, BALB-neuT transgenic breast cancer, and human-derived MDA-MB-231 xenograft models. Immune infiltration analysis showed an increase in CD4^+^ and CD8^+^ T cell populations, suggesting an improvement in the tumor immune microenvironment.

### 7.3 Epithelial ovarian cancer

Epithelial ovarian cancer (OvCa) remains the deadliest gynecological malignancy (94). A study combined the MS2 VLPs platform with deep sequencing technology (Ion Torrent) to establish a novel screening method for identifying autoantibody responses to tumor-associated antigens (TAAs) in the plasma of ovarian cancer patients, positioning TAAs as potential targets for diagnostic and/or immunotherapy strategies (95).

The study employed a random MS2 VLPs library to perform two rounds of affinity screening on plasma IgG from five patients with advanced ovarian cancer. Deep sequencing analysis of the populations, followed by bioinformatics comparison, identified a candidate peptide, DISGTNTSRA, which exhibited sequence homology with CA125 (MUC16), a widely recognized ovarian cancer biomarker. It was also confirmed that MS2-DISGTNTSRA particles induced cross-reactive anti-CA125 antibodies following mouse immunization. Further analysis of plasma samples revealed that patients with elevated levels of anti-DISGTNTSRA or anti-CA125 antibodies typically had better survival prognosis. Although the correlation between antibody levels and overall survival did not reach statistical significance, patients with normal CA125 levels or positive anti-CA125 antibodies had significantly longer median survival.

### 7.4 Ovalbumin -based tumor model

The inherent instability and low delivery efficiency of mRNA remain major barriers to the widespread application of mRNA vaccines (96). To address these challenges, a dual-promoter plasmid was designed to clone the coding sequences for MS2 VLPs and EGFP or EGFP-OVA, both carrying the pac site, into the pACYCDuet-1 vector (97). Further validation through RT-PCR and nuclease digestion assays confirmed the efficient encapsulation and protective properties of EGFP and EGFP-OVA mRNA. Stability assays showed that MS2 VLPs could be stored at 4 °C for at least three months without degradation. Cellular studies revealed that EGFP mRNA-loaded VLPs were efficiently internalized by dendritic cells, where they drove the expression of the antigen protein, demonstrating exceptional intracellular delivery and translation efficiency. In an OVA-based tumor model, MS2 VLPs encapsulating OVA mRNA significantly inhibited tumor growth. This effect was accompanied by a reduction in myeloid-derived suppressor cells, enhanced antigen-specific CD8^+^ T cell responses, and sustained humoral immunity. Additionally, when combined with alum adjuvant, the vaccine further enhanced T cell activation and prolonged antigen presentation.

## 8 Contraceptive human chorionic gonadotropin vaccine

Human Chorionic Gonadotropin (hCG) is a critical hormone for maintaining pregnancy and is considered a promising target for contraceptive vaccines (98). An ideal anti-hCG vaccine should possess high antigenic specificity to prevent cross-reactivity with structurally related hormones, including luteinizing hormone (LH), follicle-stimulating hormone (FSH), and thyroid-stimulating hormone (TSH) (99). It must also elicit a sustained immune response sufficient for efficacy without inducing irreversible infertility.

To achieve this, recombinant nanoparticles were developed using the PP7 phage platform, displaying a 10-mer peptide sequence with a 5-amino acid overlap from the C-terminal region of the hCG β-subunit (99). Additionally, the MS2 VLP platform was employed to construct a random peptide library, addressing the limitations of linear epitope presentation and complementing it with conformational epitopes. Affinity screening using the functional neutralizing antibody cPiPP successfully identified high-affinity peptides with specific binding. Immunological evaluation showed that particles displaying peptides 116 - 125, 126 - 135, and 131 – 140 successfully induced neutralizing antibodies in mice, significantly inhibiting hCG-induced uterine development, indicating they targeted functional neutralizing epitopes. In contrast, overlapping peptides such as 121 – 130 and 136 – 145 generated binding antibodies but lacked neutralizing activity, underscoring the importance of both sequence continuity and spatial conformation in neutralizing epitopes. This study combines linear peptide insertion with conformational epitope screening, using both PP7 and MS2 VLP platforms to accurately identify and validate neutralizing epitopes of hCG. This will contribute to the development of hormone-free, reversible contraceptive vaccines.

## 9 Concluding remarks

MS2 VLPs represent a promising and versatile platform for vaccine development, owing to their structural stability, efficient antigen presentation, and cost-effective production (22). Their capacity to display multiple epitopes in a highly biocompatible format makes them well-suited for both traditional infectious disease vaccines and emerging therapeutic applications, such as cancer immunotherapy (22–24, 26). Despite these advantages, several critical challenges must still be addressed before MS2 VLPs can be translated from preclinical research to clinical implementation.

A major hurdle is the rapid and stable replacement of antigenic proteins on the VLP surface. Traditional genetic fusion techniques often face limitations in flexibility and structural integrity (23, 26, 29). However, recent advancements in bio-orthogonal conjugation technologies, such as the SpyTag/SpyCatcher covalent binding system, enable modular antigen exchange while preserving particle stability (100, 101). This breakthrough opens the door to developing reprogrammable vaccine platforms capable of swift deployment against emerging infectious threats. Moreover, precise control over the stoichiometry of co-displayed antigens is essential for the development of multivalent or combination vaccines, which remains a key area for further research.

In parallel, RNA-based vaccines are transforming the field of vaccinology by offering unmatched speed, safety, and flexibility (102, 103). Unlike DNA-based or viral vector vaccines, RNA vaccines do not integrate into the host genome and are non-infectious, significantly reducing long-term genetic risks (103). Upon cytoplasmic delivery, RNA vaccines enable the direct translation of encoded antigens, demonstrating excellent biocompatibility (102, 103). However, their clinical application is hindered by poor stability during storage and inefficient delivery *in vivo (* 96). MS2 VLPs offer a potential solution by acting as “armored RNA” carriers (27, 90). Their ability to specifically package and protect exogenous RNAs, including mRNA, enhances RNA stability, shields it from degradation, and improves cellular uptake and translation. This approach could extend to other RNA-based therapeutics, such as siRNA or circular RNA, providing a unified platform for gene-based immunotherapy.

MS2-based RNA encapsidation holds significant promise for therapeutic applications, but achieving precise control over RNA cargo remains a key challenge. Pac site tagging enables selective packaging by the MS2 coat protein, but substantial co-packaging of host (*E*. *coli*) RNA has been observed, particularly with longer recombinant transcripts (40). As the RNA length increases, the proportion of host RNA incorporated also rises. To address this, a single-plasmid system was developed where pac-tagged target RNA is co-transcribed with MS2 coat protein and maturase. This system optimizes the stoichiometry between components, reducing nonspecific encapsidation (23, 40). Despite this advancement, further refinements have focused on enhancing the affinity between pac sites and the MS2 coat protein. For instance, the C5 variant, in which the fifth uridine is replaced with cytidine, has been shown to improve target RNA selectivity (42). Moreover, adding an extra copy of the pac sequence into the single-plasmid system further increases RNA packaging efficiency (104). Although pac sites play an important role in RNA packaging, recent findings suggest that selective RNA incorporation into VLPs results from cooperative interactions among multiple coat proteins and stem-loop structures distributed across the RNA molecule (105). This work provides valuable insights and opens new avenues for further exploration, suggesting that improving RNA packaging fidelity may involve additional, yet-to-be-identified elements that modulate the process. Furthermore, transitioning from *in vivo* expression systems to controlled *in vitro* assembly, using purified RNA and coat proteins, offers enhanced control over particle composition (106). This approach eliminates host RNA contamination, thus improving the homogeneity and therapeutic reliability of MS2-based VLP platforms.

Such progress in RNA cargo fidelity forms the foundation for the clinical translation of MS2 VLPs. To fully unlock their potential, however, additional development is needed in scalable biomanufacturing, product standardization, and rigorous quality control protocols (18). Efficient expression systems, high-throughput purification workflows, and reproducible RNA encapsulation strategies will be key enablers for large-scale deployment. Moreover, integration of computational tools such as artificial intelligence, systems immunology, and bioinformatics could greatly accelerate antigen design, optimize immune response predictions, and enable tailored vaccine development for broader global populations (107).

In this context, MS2 VLPs represent a structurally stable, safe, and cost-effective modular platform capable of supporting diverse biomedical applications. The synergy between SpyTag/SpyCatcher conjugation and pac site-mediated RNA encapsulation enables programmable construction of antigen–RNA co-delivery systems. In our previous work, we demonstrated that live attenuated bacterial vectors are not only effective in protein antigen delivery but also competent in transporting VLPs assembled *in vivo (* 108–110). These findings suggest that hybrid strategies such as combining MS2 VLPs with bacterial carriers could offer enhanced delivery efficiency and adaptability. As the field moves toward multivalent, rapidly deployable vaccine platforms, MS2-based technologies are well positioned to contribute meaningfully to next-generation vaccinology and global infectious disease prevention.
